# Reprogramming tumor-associated macrophages as a unique approach to target tumor immunotherapy

**DOI:** 10.3389/fimmu.2023.1166487

**Published:** 2023-04-17

**Authors:** Safir Ullah Khan, Munir Ullah Khan, Muhammad Azhar Ud Din, Ibrar Muhammad Khan, Muhammad Imran Khan, Simona Bungau, Syed Shams ul Hassan

**Affiliations:** ^1^ Hefei National Laboratory for Physical Sciences at the Microscale, School of Life Sciences, University of Science and Technology of China, Hefei, China; ^2^ MOE Key Laboratory of Macromolecular Synthesis and Functionalization, International Research Center for X Polymers, Department of Polymer Science and Engineering, Zhejiang University, Hangzhou, China; ^3^ Faculty of Pharmacy, Gomal University Dera Ismail Khan KPK, Dera Ismail Khan, Pakistan; ^4^ Anhui Province Key Laboratory of Environmental Hormone and Reproduction, School of Biological and Food Engineering Fuyang Normal University, Fuyang, China; ^5^ School of Life Sciences and Medicine, University of Science and Technology of China, Hefei, China; ^6^ Department of Pharmacy, Faculty of Medicine and Pharmacy, University of Oradea, Oradea, Romania; ^7^ Shanghai Key Laboratory for Molecular Engineering of Chiral Drugs, School of Pharmacy, Shanghai Jiao Tong University, Shanghai, China; ^8^ Department of Natural Product Chemistry, School of Pharmacy, Shanghai Jiao Tong University, Shanghai, China

**Keywords:** tumor-associated macrophages, anti-cancer treatment, immune suppression, Metabolism, macrophage-targeting immunotherapy

## Abstract

In the last ten years, it has become increasingly clear that tumor-infiltrating myeloid cells drive not just carcinogenesis *via* cancer-related inflammatory processes, but also tumor development, invasion, and metastasis. Tumor-associated macrophages (TAMs) in particular are the most common kind of leucocyte in many malignancies and play a crucial role in establishing a favorable microenvironment for tumor cells. Tumor-associated macrophage (TAM) is vital as the primary immune cell subset in the tumor microenvironment (TME).In order to proliferate and spread to new locations, tumors need to be able to hide from the immune system by creating an immune-suppressive environment. Because of the existence of pro-tumoral TAMs, conventional therapies like chemotherapy and radiotherapy often fail to restrain cancer growth. These cells are also to blame for the failure of innovative immunotherapies premised on immune-checkpoint suppression. Understanding the series of metabolic changes and functional plasticity experienced by TAMs in the complex TME will help to use TAMs as a target for tumor immunotherapy and develop more effective tumor treatment strategies. This review summarizes the latest research on the TAMs functional status, metabolic changes and focuses on the targeted therapy in solid tumors.

## 1 Introduction

The immunosuppressive tumor microenvironment (TME), which promotes the formation and progression of tumors, is characterized by the selective survival of immunoresistant tumor types (1). Among the immune cell types found in Tmes, tumor-associated macrophages (TAMs) play a significant role. TAMs have a critical role in triggering TME evolution and promoting tumor growth (2). There is strong evidence that TAMs contribute significantly to tumor development, angiogenesis, metastasis, immune system evasion, and therapy-related side effects. TAMs, like other immune cells, can switch between several different phenotypes and functionalities in response to different stimuli. There is mounting evidence that TAMs’ “immunosuppressive and protogenic” behavior results from the reprogramming of metabolic programs that affect the development and prognosis of cancer.

Considering that tumor-associated macrophages (TAMs) are the type of immune cells that are most broadly distributed in the tumor microenvironment (TME), anti-tumor immunotherapy now considers them to be an essential target. The majority of TAMs are formed from monocytes, with some TAMs also being derived from embryos, according to the growing body of research on TAMs (3). TAM interacts with the milieu and experiences metabolic alterations in the intricate tumor microenvironment, which alters the early M1-TAM’s tumor suppressor phenotype (4). According to several reports, M1-TAM predominates in the early stages of carcinogenesis and has the ability to release inflammatory substances that can slow the growth of tumors. While M1-like tumor-associated macrophages (M1-TAMs) predominate early in tumor development, M2-like tumor-associated macrophages (M2-TAMs) take over in the tumor’s intermediate and late stages, where they can increase angiogenesis, aid in tumor cell dissemination and metastasis, and have a pro-tumor effect (5, 6). Consequently, metabolic reprogramming of M2-like TAMs, preventing the recruitment drive of mononuclear cells directly deleting M2-like TAMs in tumor tissues have emerged as critical techniques for targeted TAM immunotherapy in solid tumors (7). This article covers the current targeted therapeutic methods, examines the possibilities of targeting TAMs in tumor immunotherapy, and primarily focuses on the origin and functional flexibility of TAMs in response to the tumor microenvironment.

## 2 Origin of TAM

Tumor cells and stromal cells, including fibroblasts, blood and lymphatic vessels, and immunoreactive cells (mainly macrophages and lymphocytes), are in solid tumors in varying proportions. Extracellular matrix settings provide access to a diverse pool of bioactive chemicals in soluble form or bound to proteins. This includes many proteolytic enzymes that actively change the surrounding matrix and growth factors for tumor cells and newly generated blood vessels, chemical attractants lured to immune cells in the tumor mass, and so on.

The macrophage population is typically the largest in the tumor microenvironment (8, 9). Studies from the past have demonstrated that properly activated macrophages can kill tumor cells *in vitro*. However, TAM is still widely held to have a primary (though not necessarily) tumor-promoting activity (10). TAM develops from monocyte progenitors found in the blood (11).

Tumor-associated macrophages make up 30%–50% of the immune cell population in the majority of solid tumor tissues (12). The immune cell population is an essential component of the tumor stroma. TAMs are hence regarded as the TME’s most prevalent population of tumor-infiltrating immune cells. Experimental studies using fluorescently labeled bone marrow transplants (13) and tracer microsphere-labeled monocytes (14)support the idea that TAM are fully differentiated from monocyte progenitors that invaded tissues (15). However, an increasing number of investigations (16, 17) have demonstrated that macrophages have a dual origin, deriving from both monocytes and embryos. Furthermore, carcinogenesis and the tumor microenvironment (TME) have rethought the macrophage developmental timeline.

**Figure 1:**
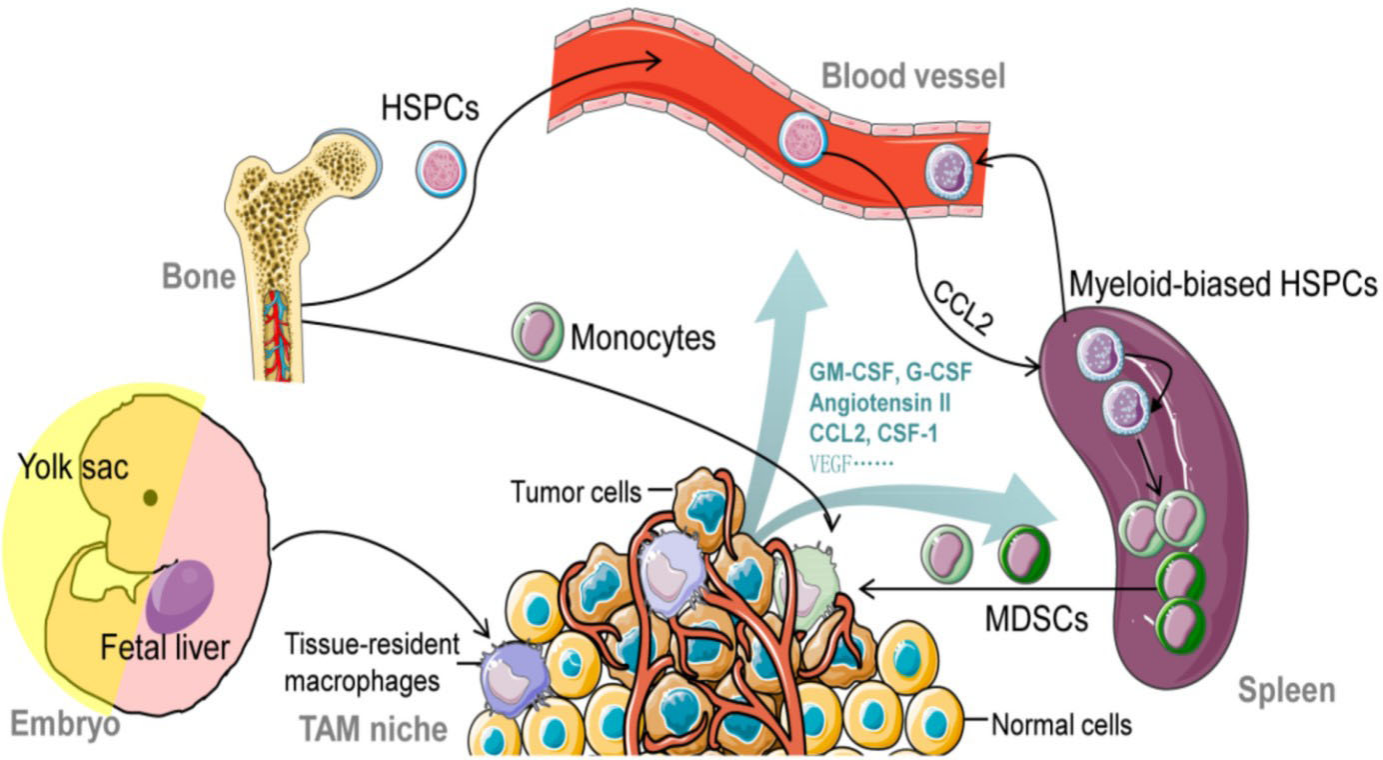
The origin of tumor-associated macrophages (TAMs). this figure was reproduced under common creative license (20).

In recent years, studies have shown that tissue-specific resident macrophages derived from embryos can also infiltrate the tumor microenvironment in specific tumor tissues, and TAM can be directly differentiated from locally resident macrophages. Cellular monocytes or embryonic precursors that seed peripheral areas and sustain themselves throughout the host’s life are the sources of tissue macrophages (18, 19). However, this origin cannot maintain the amount of TAM in tumor tissues, and its functional effect is still unclear (7). Bone marrow mononuclear cells in peripheral circulation are still the most crucial source of TAM accumulation. In disease states, tissue-resident macrophages and circulating inflammatory monocytes are recruited to the tumor periphery and develop into M0-TAMs under the recruitment of multiple chemokines (CCL2 and CCL5) and cytokines (CSF-1 and VEGF family members) (**Figure 1**). There are two main trends in the development of embryonic or monocyte-derived macrophages in specific tumor tissues: (1) Embryonic or monocyte-derived tissue-resident macrophages may undergo phenotypic or activation changes during carcinogenesis, which are called tissue-resident tumor-associated macrophages (TRTAMs). (2) The differentiated monocytes are influenced by the microenvironment during tumor growth, forming tumor-induced tumor-associated macrophages (tiTAMS) (21). trTAM and tiTAM can exist in the same specific tumor tissue. trTAM is the predominant TAM in the early tumor stage, while tiTAM is the predominant TAM in the late tumor stage (21). Therefore, the origin of TAM may be related to the type and stage of the tumor, but further research is needed.

The construction of various gene-edited mouse tumor models (22) and the development of lineage tracing technology have further revealed the origin and development of TAM and the differences in tissue distribution. For example, the Lewis lung cancer model of nonsmall-cell carcinoma (NSCLC) confirmed that TAMs are both embryonic and monocyte-derived (23). Spontaneous mammary gland carcinoma mainly exists in the TME of mice with two subsets of macrophages (CD11bhighF4/80 low MHC - IIhigh and CD11blowF4/80 high MHC); among them, CD11blowF4/80highMHC-IIlow can proliferate in situ (24), while embryo-derived tissue-resident macrophages have self-renewal ability, indicating that the source of TAM in breast cancer is both mononuclear cell infiltration and *in situ* proliferation of tissue-resident macrophages (25, 26).

## 3 Functional plasticity and metabolic changes of TAM

### 3.1 Functional plasticity of TAM

Researchers have long hypothesized that TAM can be split into two distinct polarization states, one characterized by “classical activation” (M1) and the other by “selective activation” (M2). Classically activated M1-like TAM is a tumor-associated macrophage that inhibits tumor growth by secreting pro-inflammatory cytokines and tumor necrosis factors. M2-like TAMs promote tumor development by altering the matrix, participating in phagocytosis, and releasing angiogenic factors (27).. Researchers have observed that using solely M1 and M2 polarization to split the functional state of TAM in complicated TME is extremely severe, and that TAM that is really polarized into two states is quite rare, thanks to the advent of single-cell technology. Neoplastic cells influence TAM’s differentiation and functional direction in the tumor microenvironment, and TAM, in turn, expresses many protumoral characteristics, such as the production of growth factors and matrix-proteases, stimulation of angiogenesis, and inhibition of adaptive immunity. As it has been discovered that a subset of TAMs can express M1 and M2-related marker genes simultaneously in glioma (28), breast cancer, and lung cancer (29), it is important to better characterize the related molecular markers of different types of TAMs. HLA-DR, pSTAT1 and iNOS are all prominent markers in human samples associated with M1 TAMs. CD206, CD204, and CD163 were shown to be highly expressed on M2-type TAMs due to the presence of macrophage scavenger receptors (CD204 and CD163) and mannose-receptor-1 (CD206).

There are two types of macrophages: M1 macrophages, activated in a traditional fashion, and M2 macrophages, stimulated in a more atypical fashion (30). High levels of transcriptional activator 1 STAT1 and nitric oxide synthase 2 NOS2 expression by M1 macrophages are associated with their role in the acute pro-inflammatory response, which includes removing invading pathogens and germs and increasing T-cofactor 1 (Th1) type immunity (31). However, ongoing M1 macrophage activity in inflammation may harm the tissue and hinder wound healing (32). Instead of maintaining an inflammatory response, as M1 macrophages do, M2 macrophages produce huge amounts of anti-inflammatory cytokines. Angiogenesis, fibrosis, and Th2 immunity are all aided by M2 macrophages, which are otherwise primarily involved in tissue repair and phagocytosis (33).

There are four distinct subtypes of M2 macrophages, each characterized by a unique set of responses to stimulatory chemicals and microenvironments (34). Previous research indicates that M2a macrophages promote healing and fibrosis by secreting IL-4 and IL-13 and expressing mannose-receptor C-1 (MRC1)/distinction cluster 206 (CD206) and fibronectin (35). M2b macrophages express C-C chemotactic factor ligand 1 (CCL1) and tumor necrosis factor ligand superfamily member 14 (TNFSF14) at high levels and secrete many anti-inflammatory Anti-inflammatory IL-10 production, and efficient apoptotic cell phagocytosis were hallmarks of M2c macrophages, which also overproduced Mer receptor tyrosine kinase (MerTK) (30, 36). High amounts of IL-10, TGF-, and VEGF expression by M2d macrophages make them a significant inflammatory component of tumor tissues, encouraging angiogenesis and cancer spread (37). Numerous research have looked into whether the M2 subtype is present in disorders connected to macrophages, including cancer, atherosclerosis, and kidney disease (38–40). While our knowledge of macrophage plasticity and function has come a long way, the molecular features of the four M2 subcategories (M2a, M2b, M2c, and M2d) must be thoroughly investigated. As a result, it is useful to forecast the potential regulatory effects of the four M2 subtypes using quantitative proteomic research.

The term “activation” is preferable to “polarization” when referring to TAM since TAM is not a true macrophage-polarized population. Thanks to advances in single-cell mRNA sequencing (scRNAseq), scientists can now further categorize TAM activation types beyond the conventional M1/M2 polarization model. It is possible to expand the “spectral paradigm” of macrophage activation to include at least nine groups (41).

Macrophages play a multifaceted role in both acquired immunity and innate. Macrophages are multi-purpose cells that may digest and present foreign antigens, and they also function as integrators and transducers of a wide variety of biochemical signals (42). According to recent research on macrophage transcriptome responses to various stimulating chemicals, numerous unique mRNA co-expression modules exist (41). The M1 and M2 programs, called after the products that Th1 and Th2 cells, respectively, produce, are two widely acknowledged forms of macrophage response (43). The M1 response, also known as classical activation, is frequently generated *in vitro* by administering lipopolysaccharide (LPS) plus interferon-γ (IFN-γ) to cells. (bacterial cell wall components and TLR4 agonists). Inflammatory cytokines, including TNF-α and IL-1β, are produced as part of the M1 phenotype (44). The metabolic metamorphosis of M1 macrophages also involves glycolysis and the release of free radicals. Treatment with interleukin (IL)-4 and interleukin (IL)-13 causes a reaction known as the M2 response (or substitution activation) in mice, which is characterized by elevated levels of CD206, scavenger receptor, and arginase 1. The M2 reaction was further categorized as the M2a response once different types of replacement activation were discovered. The full recognition of these modeled reactions might only be possible *in vitro*. However, M1- and M2-like symptoms can be quickly identified physiologically.

Activated M1 and M2 macrophages display distinctive transcriptional and secretory characteristics. The STAT1 and IRF pathways were activated in response to M1 activation, while the STAT6 pathway was activated in response to M2 activation (45). Both Stat1 and Stat6 activation are counteracted by activation of the opposite route in mouse macrophages, where the former is induced by interleukin 4 (IL-4) and the latter by STAT1 (46). The expression of the FcR on the surface of human monocytes that IFN -γ regulates- was also decreased by co-stimulation with IL-4. Despite signs of mutual inhibition, the body’s cells were shown to express markers for both the M1 and M2 phenotypes. This overlap in expression may indicate that the M1 and M2 programs are being activated in tandem (47). A similar development was shown in T cells, where CD4+ T cells underwent mixed culture conditions and developed into IFN-gamma-secreting Th1 lymphocytes or IL-4-secreting Th2 cells, resulting in a controlled continuous somatic destiny. This finding is consistent with the hypothesis that macrophages, like many other cell types, make decisions about their fate using mutual inhibitory, self-activating (MISA) networks, as shown by computational modeling (48).

These findings demonstrate that macrophages undergo reprogramming by mixed activation signals that depend on the initial polarization state and stimulus dose and give evidence for a phenotypic continuity in macrophages through the analysis of phenotypic markers on the single-cell level.

TAM is in a continuous transition state between the M1 and M2 types, with the ratio of each cell type depending on the type and concentration of signals in the tumor environment, The types and concentrations of different signals in the tumor environment determine the proportion of each form (49). Tumor-associated micro environmental interferon (IFN) interacts to the interferon receptor expressed on TAM, triggering the JAK-STAT signaling cascade and leading to the production of several pro-inflammatory cytokines (IL-1, IL-6, and NOS2) (50). It was encouraged for M0-TAM to become M1-TAM activated. Through the action of myeloid differentiation factor 88, moderators such as lipopolysaccharide (LPS) have the ability to detect Toll-like receptors (TLR) that are located on the surface of TAM (myeloid differentiation factor 88, TAMs). Myeloid differentiation primary response gene 88 (MYD88) mediated intracellular kinase activation and activated the critical transcription factor NF-B to control the expression of pro - inflammatory genes and spur M1-TAM activation. In response to danger signals, M1-like TAM significantly influenced the production of thiamine pyrophosphate (TPP), lipopolysaccharide (LPS), tumor necrosis factor (TNF), or interferon- by recruiting and activating cells of the adaptive immune system. These cells, in turn, release high levels of TNF, nitric oxide synthase 2 (NOS 2), reactive oxygen species (ROS), and cytokines IL-12 The LPS-induced activation of M1-TAM can be inhibited by lipoic acid (LA), which causes the engagement of TAM to switch to M2.

In the presence of high levels of IL-10, IL4, IL-13, prostaglandin (PGE), and glucocorticoid (GC) in the TME, the polarization of M0-TAM cells to M2-like TAM is facilitated by a cascade of physiological and biochemical changes triggered by the binding of M0-TAM surface receptors to these substances. High amounts of VEGF and IL-10 in tumorigenic M2-like TAMs stimulate angiogenesis and speed up the immunological escape of tumor cells. In addition to their role in immunosuppression and tumor growth promotion, M2-like TAMs can release a wide range of cytokines that promote tumor cell proliferation and survival, including epithelial growth factor (EGF), platelet-derived growth factor (PDGF), and transforming growth factor-β (TGF-β). However, with the development of tumor, the type and concentration ratio of cytokines in TME will change, and the activation phenotype of TAM will also change dynamically and play different functional roles. Tumor-associated microenvironmental (TAM) surface indicators are strongly correlated with tumor type and spatiotemporal stability (51). Consequently, the nature of the intermediate state during TAM activation and the nature of the stimulator driving this activation are both unknown (**Figure 1**).

Macrophages that tested positive for CD204 and CD206 were also strongly linked to a worse prognosis for patients with lung cancer, advanced pTNM staging, and lymph node metastases (52). As a result, CD204 and CD206 are considered helpful biomarkers of TAM activation. There is evidence that the Class A scavenging receptor CD204 plays a role in both the detection of pathogens and the development of atherosclerosis. Several tumor-associated macrophages (TAM) that are positive for CD206 are correlated with poor prognosis in patients with prostate cancer (53).

Tumor-associated macrophages (TAMs) release various angiogenic growth factors, with epidermal growth factor (EGF) being the most effective source of EGF in the tumor microenvironment (54). Due to its role as a chemokine in the cancer microenvironment, EGF hastens metastasis by increasing tumor cell motility and invasiveness (55). Additionally, EGF encourages the growth of breast cancer cells *in vitro*.

### 3.2 Metabolic changes of TAM

Emerging research indicates that TAM’s metabolic activity, such as glucose, lipid, and amino acid metabolic activity (56), also alters as a result of M2-TAM’s secretion of immunomodulatory molecules during activation, which can promote metabolic alterations in tumor cells (57). The two factors interact to create new functional phenotypes.

In the two-well co-culture paradigm of thyroid tumor cell lines and monocytes, monocyte-derived thyroid antigen antibodies were isolated from healthy participants. Increased glycolysis was one of the most notable features of the metabolic transcriptome that was detected after activation of the protein kinase B/mammalian receptor of these TAMs’ rapamycin (AKT1/mTOR) system. Thyroid cancer samples taken examined by immunohistochemistry showed that M2-TAM had elevated expression of glycogen synthesis enzymes and lactate sensors (58). M2-TAM not only showed altered glucose metabolism, but also altered lipid metabolism, namely an increased ability for fatty acid production, absorption, and storage. In particular, immunological dysfunction is brought on by the aberrant metabolism of arachidonic acid in TME, which occurs when free arachidonic acid is converted by cyclooxygenase (COX) and sometimes lipoxygenase (LOX) into prostaglandin E2 (PGE2). PGE2 and HETE can enhance M2-like activation of TAM, and PGE2 can stimulate M2-like engagement of TAM. Numerous clinical and pharmacological investigations have demonstrated that the synthesis of COX2 and PGE2 is elevated in many different forms of cancer, including lung cancer, colorectal cancer, thyroid cancer, and prostate cancer. This results in M2-like TAM activation, suppression of T and B lymphocyte proliferation, and reduction of their killing capabilities, all of which speed up the formation and growth of tumors (59, 60). The function of TAM can potentially be impacted by amino acid metabolism. Arginase-1 (ARG1) expression is upregulated in M2-TAM in several mouse tumor models, while glutamate transport and other metabolic genes are upregulated in M1-TAM in mouse glioma (43). These findings imply that the urea cycle might be crucial to TAM operation. To fully understand TAM function and its method of action, however, we need to better understand the connection between each metabolic alteration and TAM.

## 4 The role of TAM in different types of tumors

Metastatic tumor and advanced cancer samples showed that a greater number of M2-TAM was related with a poor prognosis for individuals with a wide range of solid tumors, such as thyroid, lung, gastric, breast, prostate, uterine, brain, and liver cancers (61–63). TAM is a cell population with significant heterogeneity. M2-TAM activation has been linked to immunosuppression, promotion of angiogenesis and lymphangiogenesis, regulation of tumor cell metabolism and proliferation, and induction of drug resistance (64).

**Figure 2:**
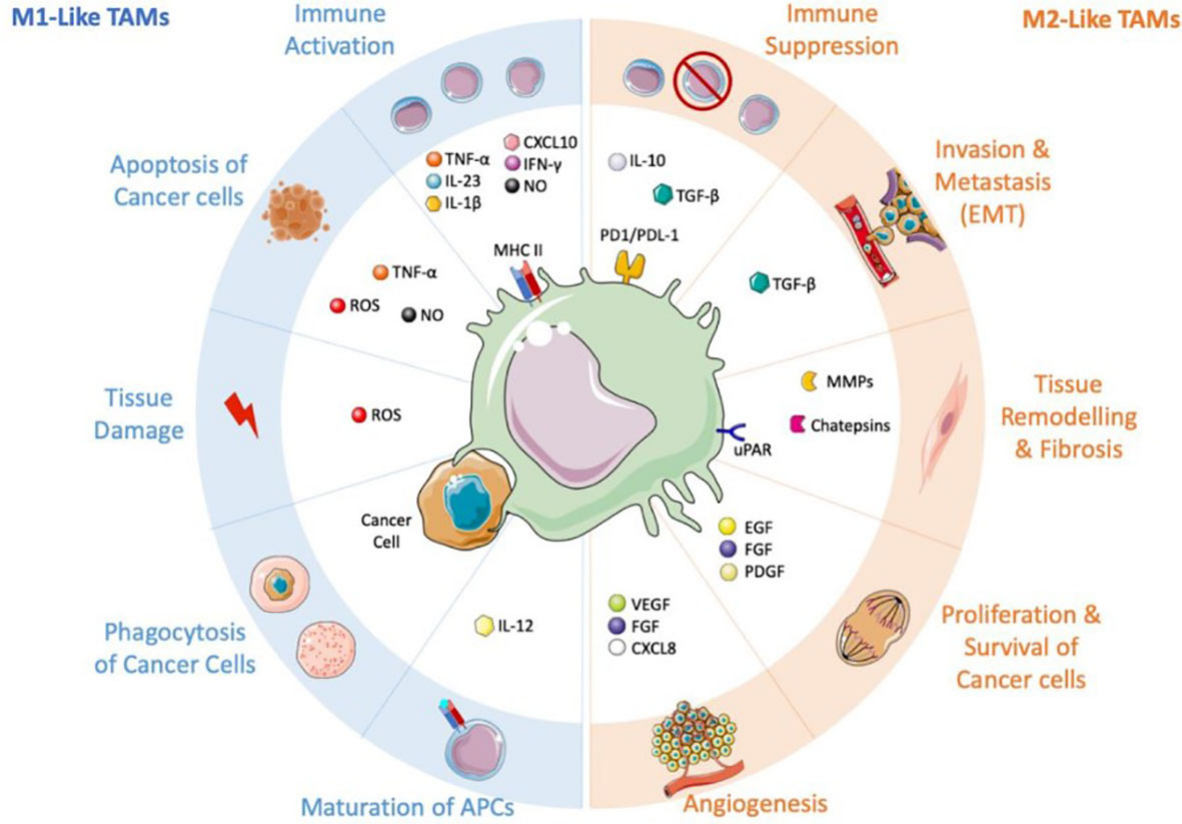
TAMs and their conflicting influence on the tumor microenvironment. this figure was reproduced under common creative license (65).

Macrophages seen in tumors are the primary immune cells in solid tumors, making up as much as half of the tumor’s total mass. Both tissue-resident macrophages and circulating monocytes can produce them. Rapid recruitment of circulating inflammatory monocytes to the site of tumor growth can be achieved by specific signaling molecules such as CCL2, CSF-1, mediators, and complement components (in particular C5). Tumor-associated macrophages (TAMs) can potentially originate from resident macrophages that were already present in the tissue before the malignancy developed. In response to various stimuli released by the tumor microenvironment, TAMs can either adopt an immunosuppressive, proneoplastic phenotype (shown in **Figure 1**) or a proinflammatory, anticancer phenotype (**Figure 2**) **(** 66–68). Consequently, macrophages have been shown to play a dual function in the onset and progression of many different cancers (69, 70), and their abundance and polarisation status are associated with patient prognosis. Overall survival and metastasis-free survival are improved when they are present in various tumor forms, including osteosarcoma and esophageal cancers (71–73). In contrast, macrophages are linked to a poorer prognosis in some malignancies, especially when paired with a lack of CD8+ cells (74–77), the type of lymphocyte responsible for destroying tumor cells.

Therefore, TAMs with M2-like traits may be viewed as the immune system’s “corrupted policemen” (2) because they are commonly linked to and accountable for the disease’s poor prognosis. Through the release of signaling molecules including transforming growth factor beta (TGF-),macrophage colony-stimulating factor (M-CSF), vascular endothelial growth factor (VEGF),inflammatory cytokines or chemokines (IL-10, IL-6, and CXCL-8) (78–80), and extracellular vesicles (EV) with immunosuppressive qualities (81), they are involved in the beginning and advancement of the tumor.

deleted 3 different tummer types

## 5 Expression of TAMs in a variety of tumors

It is now generally known that massive concentrations of TAMs with an M2-like pro function are frequently linked with poor clinical outcomes. This is because the features of macrophages that were discussed in the paragraph before this one was taken into consideration. On the other hand, TAMs that polarize toward an M1-like pro-inflammatory character tend to have a better diagnosis and longer survival times (**Table 1**) **(** 88, 98–101). The characteristics of macrophage polarization in various tumor types are discussed below.

**Table 1:** TAM and marker prognosis in several human different cancers.

Tumor Type	Markers of TAMs	Prognostic Impact	References
Mesothelioma	CD68	Bad	(82)
	CD163	Bad	(83)
Pancreatic cancer	CD68, CD204	Bad	(84)
Colorectal cancer	CD68	Good	(85)
	Wnt5a, CD68	Bad	(86)
Lung cancer (NSCLC)	CD68/iNOS (for M1); CD68/CD163 (for M2)	Bad	(87)
	CD68	Good	(88)
Breast cancer	CD68, CD163	Bad	(89)
	iNOS	Good	(90)
	CD163	Bad	(91)
Glioma	IBA, CD204	Bad	(92)
	CD68, CD163/AIF ratio	Bad	(93)
	CD68, CD163, CD204	Bad	(94)
	CD163/CCL3 ratio	Bad	(95)
Bladder cancer	CD68	Bad	(96)
Melanoma	CD68, CD163	Bad	(97)

**Table 1**. TAM and marker prognosis in several human different cancers

### 5.1 Targeted immunotherapies with TAM

The tumor has become one of the most important causes of harm to human life. Targeted tumor immunity has had great potential in anti-tumor therapy in recent years. In addition to targeting normal T cells and B cells, TAMs have gradually become a popular target for tumor immunotherapy. With a better understanding of the interaction between TAM, TME, and tumor cells, many anti-tumor therapy-related explorations have been carried out in targeting TAM, including Inhibitory monocytes recruited to tumor tissue, directly removing M2 from TAM, inhibiting the conversion of M1-like anti-tumor-TAM-to-M2-like-tumor-promoting TAM, and promote the conversion of M2-like TAM to M1 TAM, as well as metabolic reprogramming of TAM (102, 103).

**Figure 3:**
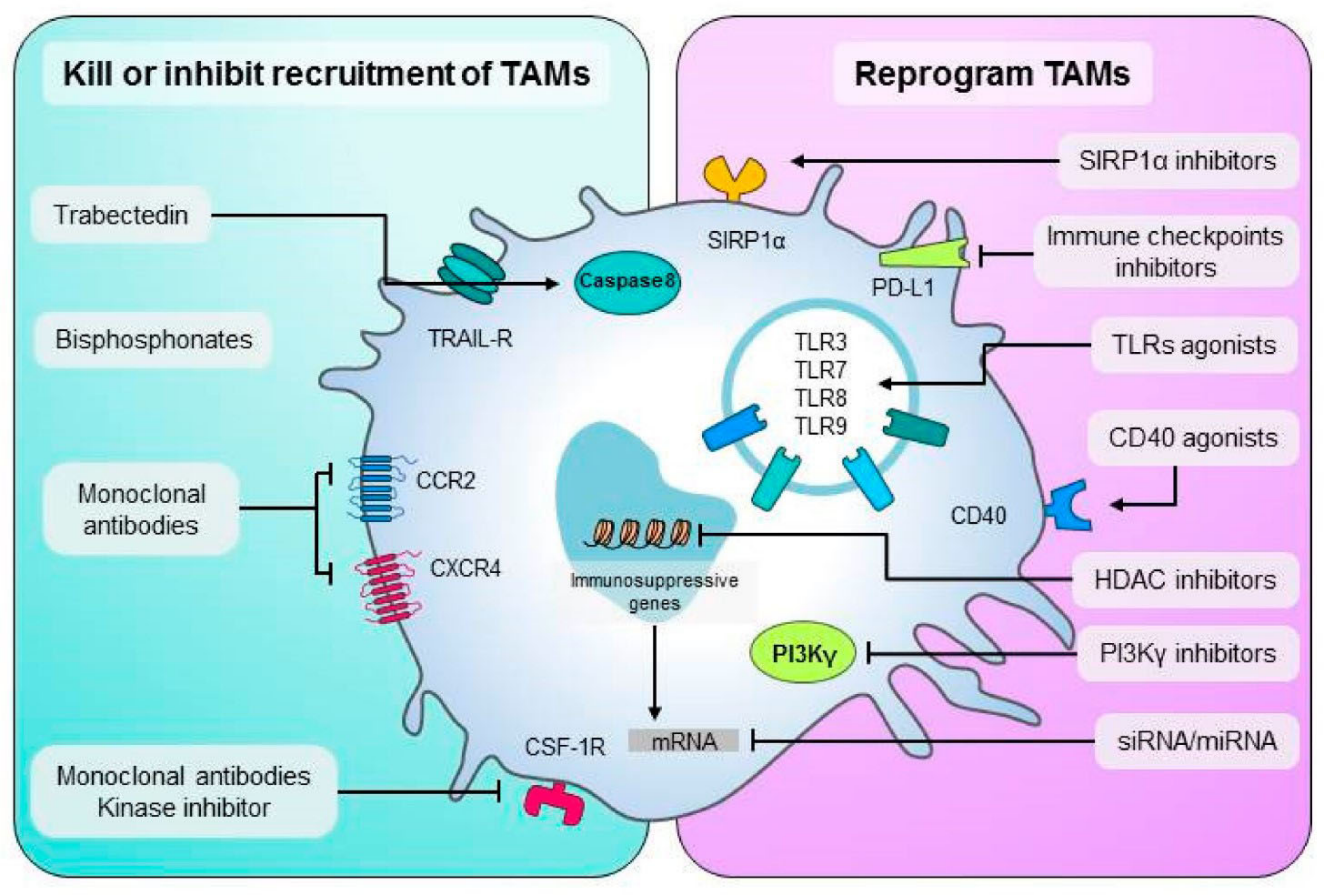
An overview of TAMs-targeting treatment approaches. Left are methods to destroy macrophages or prevent their recruitment in malignancies. To recruit additional macrophages to the tumor, monoclonal or kinase inhibitors disrupt the CSF-1/CSF-1R, CCL2-CCR2, or CXCL12/CXCR4 axis. Methods to convert TAMs into anti-tumor M1 effectors are shown on the right. TAM activation is achieved through the use of CD40 monoclonal agonist antibodies or Toll-like receptor agonists. TAMs can be targeted by SIRP1α inhibitors and immune checkpoint ligand mAbs like PD-L1. this figure was reproduced under common creative license (104).

There are several methods that focus on TAMs to boost anti-tumor immune responses, and a summary of these methods is shown in (**Figure 3**).

### 5.2 Inhibition of TAM precursor recruitment

One strategy to target TAMs is to inhibit the recruitment of TAM precursors, that is, to prevent monocytes from being recruited into tumor tissues and their activation into M2-like TAMs.

#### 5.2.1 Blocking the CSF-1/CSF-1R signaling pathway

Colony-stimulating factor-1 (CSF-1) is a well-established tumor stimulator due to its ability to entice and direct the migration of mononuclear cells to tumor locations. This migration can be inhibited by blocking the CSF-1/CSF-1R signaling pathway. Additionally, it encouraged M2-TAM activation. The monocyte-specific tyrosine kinase receptor colony-stimulating factor 1 (CSF-1R) responds to colony-stimulating factors. CSF-1R dimerizes after binding to CSF-1 or IL-34 and sends out a signal. Last but not least, it encourages M2-TAM survival, migration, and proliferation (105). Several antibodies and inhibitors exist that block the kinase activity of CSF-1 receptors and the CSF-1 cytokine. Anti-CSF-1R antibodies, including RG7155, IMCCS4, and FPA008, and CSF-1R inhibitors, such PLX3397, JNJ-4034627, ARRY-382, and BLZ-945, are all in the midst of clinical review right now (106, 107). In addition, the production of miR-26a, which belongs to a family of short non-coding RNA, can decrease CSF-1 production and M2-TAM engagement in primary liver cancer (108).

Using BLZ945(CSF-1R inhibitor) in a mouse glioma model reduces tumor growth and increases the number of days mice live (109). The anti-tumor effectiveness of CD8+T cells has been demonstrated to be improved and the survival duration of mice with colon cancer lengthened in other studies using a combination of PLX3397(CSF-1R inhibitor), oncogenic virus, and anti-PD-1 antibody (110). Results from clinical trials indicate that the anti-tumor effect of CSF-1R inhibitors is still modest, despite their efficacy in targeted M2-TAM treatment. Tumor-derived CSF-1 can suppress the migration of monocytes by causing cancer-associated fibroblasts (CAFs) to stop producing HDAC2-mediated chemokines that attract monocytes. This suppressive effect was reversed when the CSF-1R inhibitor was given alone, leading to a dramatic upsurge in monocyte recruitment to the TME. The good news is that CSF-1R inhibitors can be used in tandem with other therapeutic approaches to fix this issue.

#### 5.2.2 Block CCL2/CCR2 interaction

Tumor cells recruit monocytes expressing its receptor CCR2 by releasing chemokines such as CCL2 and activating them into M2-like TAM. M2-TAM plays avital role in promoting tumor cell invasion and metastasis. Therefore, targeting CCL2/CCR2 is a feasible anti-tumor treatment strategy. Studies have shown that blocking CCL2/CCR2 interaction can inhibit the recruitment and activation of M2-TAM and significantly reduce the incidence of tumors: blocking CCL2 by crumb (CNTO88) can inhibit the growth of prostate cancer cells (111, 112); PF-04136309(CCR2 inhibitor) can act on CCR2+ monocytes, prevent the recruitment and migration of M2-TAM, and further enhance anti-tumor immunity (113). PF-04136309 also shows good tolerance in the clinical treatment of advanced pancreatic cancer. A combination of CCR2 inhibitors with anti-PD-1 drugs can treat cutaneous T-cell lymphoma (114). However, in metastatic pancreatic cancer, the safety of the PF-04136309 inhibitor has been raised when combined with other therapeutic agents such as paclitaxel. In the complex environment of TME, the addition of other chemokines or cytokines is most likely to compensate for the loss of CCL2/CCR2 through a compensatory effect, so the clinical tolerance of CCL2/CCR2 inhibitors combined with other drugs still needs to be further evaluated.

### 5.3 Direct clearance of M2-TAM

The elimination of M2-TAM is the most direct means to combat its tumor-promoting effect in immunotherapy. Combining radiotherapy, chemotherapy and targeted immunotherapy is an effective method to eliminate M2-TAM in clinical treatment. TAM can phagocytose clodronate liposome (Clo-LipoDOTAP) in the body, which releases clodronate and is metabolized to non-hydrolyzable ATP analogs, leading to mitochondrial respiratory chain blockade and showing cytotoxicity against M2-TAM. scientist used Clo-LipoDOTAP to treat solid tumors in mice and found that it could eliminate M2-TAM in tumor tissues and significantly improve the survival rate of mice (115, 116). Another researcher developed a new type of clodronate liposome that can eliminate M2-TAM in B16/F10 subcutaneous tumors and significantly slow the growth of primary tumors (117, 118). M2pep is a unique pro-apoptotic peptide that preferentially binds to and kills mouse M2-TAM *in vivo*. It has a low affinity for other leukocytes. Without anticancer drugs, M2pep alone can selectively reduce the M2-like TAM population, thereby improving the survival rate of tumor-bearing mice (119).Trabeculomycin (ET743, Yondelis) is a natural alkaloid derived from Caribbean tunica. It can induce DNA double-strand breaks in M2-TAM, break the cell cycle, and has potent anticancer properties (120, 121). It has also received marketing approval to treat ovarian cancer and soft tissue sarcoma (122). Some targeted therapy strategies based on the elimination of M2-TAM include using toxin-conjugated monoclonal antibodies and the formation of cytotoxic T lymphocytes to deplete M2-TAM. This targeted therapy is accurate and efficient, but the problem of toxic side effects still needs to be overcome.

### 5.4 Targeted M2-TAM reprogramming

TAMs are highly changeable, displaying an M1-like phenotype during the early stages of malignancies and transitioning to an M2-like phenotype during the middle and late stages. Some TAMs in the tumor microenvironment (TME) are of the M1 type, which can identify tumor cells and trigger an immune response, whereas other TAMs are of the M2 type, which can encourage tumor development and proliferation. Consequently, converting malignant cells M2-like TAMs towards anti-tumor M1-like TAMs is another possible tumor treatment method (123).

#### 5.4.1 Restoration of TAM phagocytosis

Normal cells have the ability to express anti-phagocytic substances in a steady-state environment to evade phagocytosis and clearance by phagocytic cells (such as macrophages), or “phagocytosis checkpoint,” The exploitation of “phagocytic checkpoints” by tumor cells to circumvent immune monitoring has been demonstrated in numerous investigations (124). Therefore, finding the phagocytosis checkpoint and manipulating it could be a novel therapeutic strategy for targeting M2-TAM to destroy tumor cells. CD47 can be recognized by the signal-regulatory protein (SIRP), which then emits a signal that says, “Do not eat me.” Myeloid cells with elevated SIRP expression had low patient survival rates. CD47 antibody blocks CD47-SIRP interaction, allowing TAM to once again phagocytose tumor cells and halt tumor growth (125). Anti-CD47 antibodies can induce M1-like TAM development in tumor tissue and decrease tumor growth in a mouse model of non-small cell lung cancer (126, 127), and CD47 inhibition can switch TAM from a pro-tumor morphology to an anti-tumor phenotype in glioblastoma (128). Human lymphoid tumor, bladder cancer, and breast cancer preclinical models show that anti-CD47 antibody treatment promotes adaptive immunity and exerts anti-tumor effects *via* CD8+T cells and dendritic cells (129). To maximize the specificity and effectiveness of the anti-CD47 antibodyenclose the nanoparticles of calcium carbonate in a fibrin gel coating (130).

#### 5.4.2 Activation of Toll-like receptors

Innate immunological pattern-recognition receptors called toll-like receptors (TLRs) can be triggered by viral nucleic acid and lipopolysaccharide found in bacterial particles (131, 132). Activated TLRs effectively transform TAM into an M1-like phenotype in TME (133, 134). In order to speed up the activation of innate and adaptive immunity, agonists for Toll-like receptors (TLRs) such as TLR3, TLR4, TLR7/8, and TLR9 might be used (135). The most widely used TLRs—TLR9, TLR7, and TLR3—are activated by thiophosphate cytosine guanine oligodeoxynucleotide, imiquimod, and poly(I: C) (136). Toll-like receptor 7 (TLR7) and TLR9 (TLR9) agonist intratumoral injection increases tumor-associated monocyte infiltration and TAM phenotypic modification in a mouse model of breast cancer (137). Anti-tumor action of TAMs is enhanced in melanoma patients when treated with TLR7 and TLR8 agonists (3M-052), which also cause the modification of M2-like TAMs into M1-like TAMs (138). Only the TLR7 ligand imiquimod has been licensed for clinical usage thus far, and it has demonstrated anti-tumor effect in basal cell carcinoma, melanoma, and cutaneous metastases of breast cancer (139). Furthermore, IMO-2055, a TLR9 ligand, and imiquimod, a TLR7 ligand, have both been shown to have anti-tumor activities (140). TLR agonist-loaded nanoparticles can help to better induce TAM reprogramming. For instance, R848(TLR7/TLR8 agonist)-loaded nanoparticles favor accumulation in TAM and encourage the transition from M2-like to M1-like TAM (141). Nano gels coated with TLR agonists and long peptide antigens that activate TAM antigen-presenting activity were shown in another investigation on tumor immunotherapy resistance to transform immunosuppressive M2-TAMsinto immunosusceptible M1-TAMs. By enhancing the precision of targeted medication administration and allowing for long-term remodeling of TAM, this nanotechnology lessens the drug’s damaging side effects on unintended tissues (142).

#### 5.4.3 Phosphoinositide 3-kinase-γ expression suppression

A molecular switch is employed to inhibit both the “immunosuppressive program” and the “immune stimulating program” by inhibiting phosphatidylin-ositol-3-kinase (PI3K) (143, 144). Loss of PI3K activity in TAM (145), can increase the development of MHC-II and pro-inflammatory mediators while decreasing the expression of IL-10 and arginase, two molecules that serve to inhibit the immune system. Together, PI3K inhibitor (NVP-BEZ235) and immune checkpoint inhibitors displayed synergistic reduction of tumor growth (146, 147). Inhibition of PI3K can reduce the immunosuppressive state by transforming macrophages, which in turn reduces tumor cell proliferation and metastasis in mice models of breast cancer and ductal adenocarcinoma (148). As a result, PI3K inhibition can regulate how quickly M2-TAM switches from immune suppression to immunological activation, making immunotherapy with other checkpoint inhibitors a more potent therapeutic approach (149).

#### 5.4.4 Alternative methods to combat TAM remodeling

siRNA-based anticancer drugs have entered clinical trials, in which some siRNAs have been used to reprogram TAMs and convert them into M1-like TAMs (150). Sirna-nanoparticles with mannosylation can target TAM to regulate the NF-κB pathway, thereby inducing immune activation (151). Targeted delivery of sirNA-peptide nanoparticles to TAM significantly reduced M2-TAM in tumor tissues and improved animal survival (152). Also, it has been shown that various lncRNAs (long ncRNAs), circRNAs (circular RNAs), and miRNAs (microRNAs), such as miR-1155, miR-23b-3p, lncRNA-0243 (153), can re-program anti-inflammatory and pro-tumor M2-like TAMs into anti-tumor M1-like TAMs.

In malignancies, the absorption of nanoparticles may encourage macrophage activation. In tumor tissues, for instance, nanomaterials (Ferumoxytol) can trigger the pro-inflammatory response of M1-TAM by inducing the mRNA transcription of M1-like TAM, hence reducing tumor growth. Preventing tumor growth with intravenous infusion of iron oxide nanoparticles has been demonstrated to be beneficial in studies, and this technique has been approved for clinical treatment of early-stage breast cancer. Furthermore, M2-like TAM can be reversed using the nanomaterial polymer N-(2-hydroxypropyl) methacryloyl (154). While the aforementioned siRNA and nanoparticle-based therapy approaches to TAM modification have showed promising clinical promise, further research is needed to determine their efficacy, safety, and tolerance in the context of the complex tumor microenvironment in humans.

### 5.5 Metabolic reprogramming of TAM

The recruitment, migration, and function of TAM require energy consumption. Targeting TAM metabolism can open up a new way for immunotherapy. Rapamycin, a specific inhibitor of mTOR, can transform M2-like TAMs into M1-like TAMs by inhibiting the production of mitochondrial ROS and NLRP3 inflammasome, indicating that targeting upstream molecules of glucose metabolism will be beneficial to the anti-tumor effect of TAMs (155, 156). Some studies have found that metformin, a hypoglycemic drug, can reduce the M2-like polarization of TAM in mouse pancreatic tumor and bone sarcoid tumor models (157). 2-deoxyglucose, a competitive inhibitor of hexokinase (HK2), can specifically inhibit the glycolysis of M2-TAM in TME and destroy the tumor-promoting phenotype of M2-TAM, but the molecular mechanism remains to be studied (158). Because TAMs can become lipid-accumulating and differentiate into a pro-tumorigenic phenotype as a result of activated Caspase-1, Caspase-1 inhibitors like NCX-4016, YVAD, and VAD can rewire TAMs into an anti-tumorigenic phenotype and stop tumor growth *in vivo* (159). Using an ATP-binding transporter, tumor cells encourage cholesterol efflux from the M2-TAM membrane (ABC transporter). The ABC transporter encourages the expression of IFN-γ inhibiting M2-TAM genes (inducing M1-TAM activation). Therefore, genetic deletion of the ABC transporter or targeting of the ABC transporter prevented membrane sterol efflux from M2-TAM in mouse models of the bladder, melanoma, and ovarian cancer. However, the pro-tumor phenotype of M2-TAM was transformed into an anti-tumor phenotype (160). As one of the signature molecules characterizing the activation of M2-like TAMs, specific targeting of ARG1 in M2-TAMs may show critical therapeutic implications. Among them, L-norvaline (an inhibitor of ARG1) can inhibit the proliferation of M2-like TAM after overexpression of ARG1 *in vitro* (161). Although there is substantial evidence that targeting the metabolic processes of TAMs in mouse tumor models is effective in suppressing tumor growth, more preclinical studies are needed to determine whether reprogramming the epigenetic and metabolic networks of TAMs can be used as a means to facilitate immunotherapy.

In the cancer microenvironment, macrophages that have migrated into tumors or those found in ascites may change their metabolic rate (162). Unlike other cells, TAMs can promote cancer growth and suppress the immune system because of the unusual combinations of nutrients they require. It has been established in experimental settings that certain cancer microenvironments can foster either tumor-friendly or anti-tumor macrophage transformation through changes in metabolic pathways (163). Historically, arginine metabolism was used to divide M1 and M2 populations. Not only does arginine metabolism modify TAMs in TMEs, but so do several other metabolic processes. The polarization of TAMs into M1- or M2-like phenotypes is greatly influenced by several insoluble and soluble components of ovarian TMEs, such as the peritoneum and main site. In contrast to other solid tumors, ovarian cancer has its unique tumor microenvironment. It is common for cancer cells to break away from the original tumor and travel to the abdominal cavity, where they might produce malignant ascites (164). The ascites are the primary tumor microenvironment site, and numerous cell types and metabolites contribute to reprogramming. Malignant ascites rely heavily on macrophages. However, this process involves many different types of cells (165). Macrophage and cancer cell metabolic reprogramming overlap, which may be a decisive factor in antitumor response.

## 6 Conclusions

Multiple studies have revealed that TAMs can emerge from a wide variety of cell types, and that TME is home to a plethora of TAMs at varying stages of activation. TAMs can undergo metabolic and functional alterations because to the tumor’s unique microenvironment. A high concentration of M2-like TAM might encourage the growth and spread of tumors, giving cancer patients a bad prognosis. TAM has consequently emerged as a very effective target for targeted therapy. Evidence from both preclinical and clinical settings suggests that halting the recruitment of M2-like TAMs, or encouraging the removal and reprogramming of these cells, might effectively halt tumor progression and improve cancer patients’ prognoses. Combining TAM-targeted immunotherapy with other tumor treatments has been shown to have a more potent anti-tumor effect, and this is especially important in light of the possibility of drug resistance to TAM therapy and the possibility of cytotoxic side effects that may be produced by large reduction or overwhelming reversal of TAM.Although significant progress has been made in the treatment of TAMs, there are still numerous targets that have not been identified, or for which adequate targeted medications have not been created, and the mechanism by which several therapies play a critical role in TAMs treatment has not been elucidated. When paired with high-resolution spectrometry for precise metabolite identification, experimental methods that leverage *in vivo* tracer technologies and single-cell technology to explore the temporal and spatial dimensions of TAM metabolism can provide more precise advice for targeted TAM metabolic activities. It’s crucial to keep assessing in clinical trials whether patient groups are suited for TAM-targeted therapy or which patients are appropriate for combination therapy using TAM and other specific immune cells for illness indication, treatment, treatment, and prognosis.

### 6.1 Future prospective

It will be possible to comprehend tumors TMEs better and learn more about them by investigating the many states of TAMs utilising high-throughput next-generation technologies, There has been a need for mechanical knowledge of the molecular processes necessary for these adjustments; as a result, the search for treatment techniques that affect metabolic pathways is only satisfactory if all therapeutic targets are investigated. Current programme decisions will be strengthened, and new directions for precision immuno-oncology will be opened by considering TAMs as crucial participants. Macrophages may also help in tumor therapy, assisting in avoiding and controlling treatment-related side effects in cancer cases.

## Author contributions

SK and MUK writing original paper. MA collecting data of articles. IK and MIK drawing diagrams. SB and SH supervision, project administration and funding acquisition. All authors contributed to the article and approved the submitted version.
